# Coronary Artery Disease in Patients Presenting With Acute Ischemic Stroke or Transient Ischemic Attack and Elevated Troponin Levels

**DOI:** 10.3389/fneur.2021.781553

**Published:** 2022-01-13

**Authors:** Mathieu Kruska, Anna Kolb, Christian Fastner, Iris Mildenberger, Svetlana Hetjens, Maximilian Kittel, Kathrin Bail, Michael Behnes, Ibrahim Akin, Martin Borggrefe, Kristina Szabo, Stefan Baumann

**Affiliations:** ^1^Department of Cardiology, Angiology, Haemostaseology and Medical Intensive Care, Medical Faculty Mannheim, University Medical Centre Mannheim, European Center for AngioScience (ECAS), German Center for Cardiovascular Research (DZHK) Partner Site Heidelberg, Heidelberg University, Mannheim, Germany; ^2^Department of Neurology, Medical Faculty Mannheim, Mannheim Center for Translational Neurosciences (MCTN), Heidelberg University, Mannheim, Germany; ^3^Institute of Medical Statistics and Biometry, Medical Faculty Mannheim, University Medical Center Mannheim, University of Heidelberg, Mannheim, Germany; ^4^Institute for Clinical Chemistry, Medical Faculty Mannheim of Heidelberg University, Mannheim, Germany

**Keywords:** acute ischemic stroke, coronary artery disease, troponin, myocardial ischemia, transient ischemic attack

## Abstract

**Background:** There is little information concerning the invasive coronary angiography (ICA) findings of patients with acute ischemic stroke (AIS) or transient ischemic attack (TIA) with elevated troponin levels and suspected myocardial infarction (MI). This study analyzed patient characteristics associated with ICA outcomes.

**Methods:** A total of 8,322 patients with AIS or TIA, treated between March 2010 and May 2020, were retrospectively screened for elevated serum troponin I at hospital admission. Patients in whom ICA was performed, due to suspected type 1 MI based on symptoms, echocardiography, and ECG, were categorized according to ICA results (non-obstructive coronary artery disease (CAD): ≥1 stenosis ≥50% but no stenosis ≥80%; obstructive CAD: any stenosis ≥80% or hemodynamically relevant stenosis assessed by FFR/iwFR).

**Results:** Elevated troponin levels were detected in 2,205 (22.5%) patients, of whom 123 (5.6%) underwent ICA (mean age 71 ± 12 years; 67% male). CAD was present in 98 (80%) patients, of whom 51 (41%) were diagnosed with obstructive CAD. Thus, ICA findings of obstructive CAD accounted for 2.3% of patients with troponin elevation and 0.6% of all stroke patients. The clinical hallmarks of myocardial ischemia, including angina pectoris (31 vs. 15%, *p* < 0.05) and regional wall motion abnormalities (49 vs. 32%, *p* = 0.07), and increased cardiovascular risk indicated obstructive CAD. While there was no association between lesion site or stroke severity and ICA findings, causal large-artery atherosclerosis was significantly more common in patients with obstructive coronary disease (*p* < 0.05).

**Conclusion:** The rate of obstructive CAD in patients with stroke or TIA and elevated troponin levels with suspected concomitant type I MI is low. The cumulation of several cardiovascular risk factors and clinical signs of MI were predictive. AIS patients with large-artery atherosclerosis and elevated troponin may represent an especially vulnerable subgroup of stroke patients with risk for obstructive CAD.

## Introduction

Major adverse cardiac events are a leading cause of death within the first weeks after acute ischemic stroke (AIS) (1). Common risk factors contribute to the high co-prevalence of cardiac disease in patients with AIS. Coronary artery disease (CAD) is reported in every fourth patient diagnosed with AIS without a history of CAD, while the long-term risk of myocardial infarction (MI) after a transient ischemic attack (TIA) or AIS is particularly high in patients with known CAD (2, 3). Several studies have investigated AIS and the mid- and long-term risk for MI (3–5); however, little is known about coronary artery findings in patients with cerebral ischemia and suspected concomitant acute myocardial ischemia.

The assessment of troponin (TnI) levels at presentation in the emergency department is recommended by current AHA and ASA guidelines for the early management of patients with AIS (6). Elevated TnI levels are detected in highly sensitive assays in 20 to 55% of patients after an AIS or TIA and are considered as a predictive marker of an adverse clinical outcome (7). Apart from acute MI, conditions such as non-ischemic acute myocardial injury or reduced renal function may lead to elevated TnI levels (8, 9). Stroke itself may cause acute myocardial injury and myocardial dysfunction due to autonomic dysregulation, and its severity and site have been linked to TnI elevation (10).

A coronary culprit lesion as an angiographic finding is less common in patients with AIS and TnI elevation than in patients with a non-ST-elevation MI in the context of acute coronary syndrome (11). Obstructive CAD can cause either type I or II MI (12), while a coronary culprit lesion precipitated by atherosclerotic plaque is the most frequent pathomechanism of type I MI. Hemodynamically relevant coronary stenosis may also play a crucial role in ischemic myocardial damage via a mismatch between oxygen supply and demand, which is classified as type II MI. Both MI types lead to acute myocardial ischemia, but only type I is likely to benefit from urgent revascularization.

The various causes of ischemic and non-ischemic mechanisms for TnI elevation and the relevance of distinguishing between type I MI, type II MI, and acute myocardial injury in the clinical setting of patients with AIS and TIA, highlight the dilemma of the diagnostic workup for suspected MI. Additionally, an impaired neurological prognosis, comorbidities, and the risk of secondary intracerebral hemorrhage illustrate the challenge of determining the indication for ICA and revascularization. While angina pectoris remains a defining feature in the diagnosis of acute coronary syndrome, disabling neurological symptoms may affect the ability to report symptoms, hampering the identification of patients at risk for acute myocardial ischemia. Regional wall motion abnormalities or ischemic-like ECG changes are frequent in AIS, and lowering sensitivity for MI may refer to the premorbid cardiac status or to a stroke-induced heart injury (13, 14).

The data available for analysis of patients with AIS and TIA and suspected MI are scarce. In this study, the association between characteristics of patients with AIS or TIA with elevated troponin at hospital admission and ICA findings is analyzed using real-world data to improve the risk stratification and diagnostic cardiac workup.

## Materials and Methods

### Design and Patient Population

**Figure 1:**
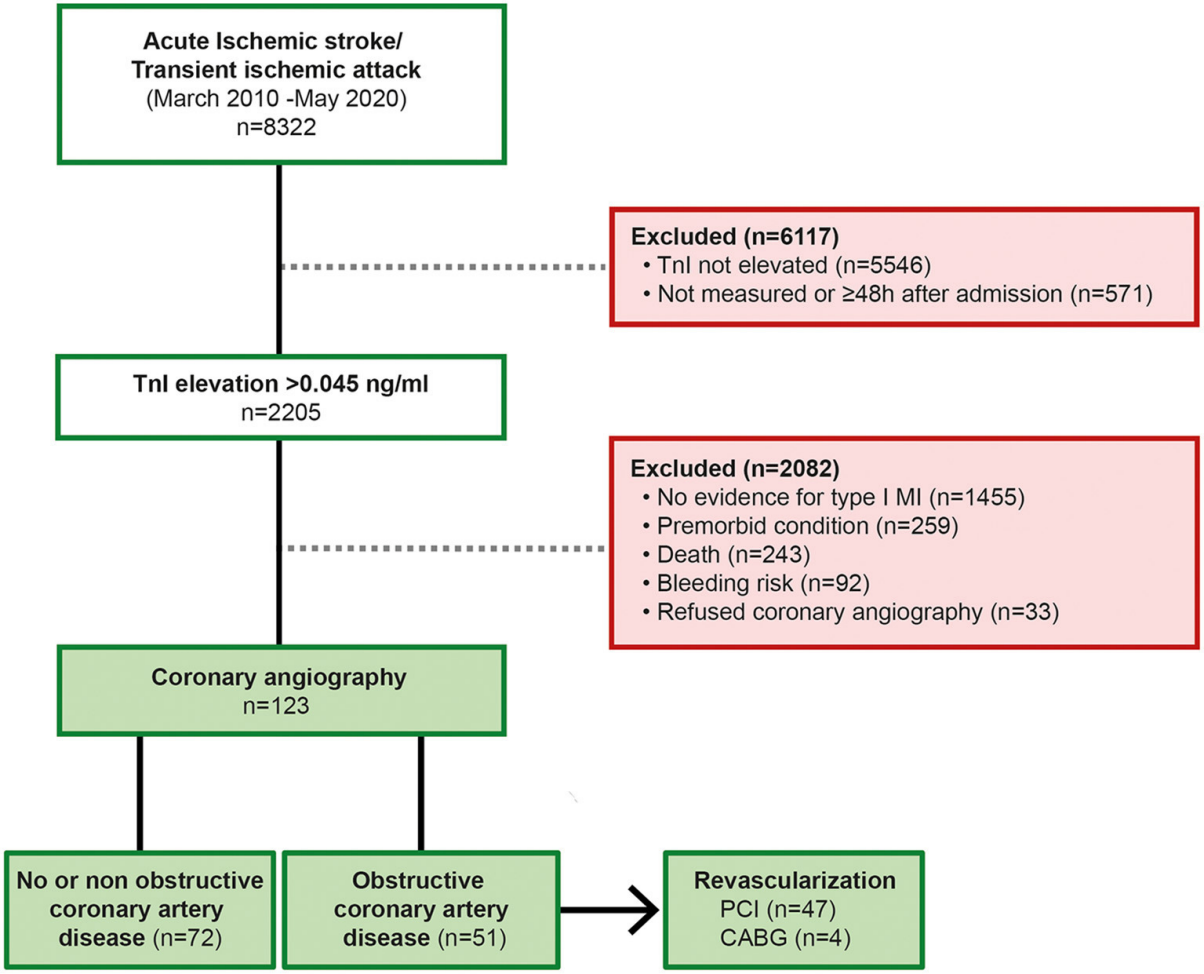
Flowchart illustrating the enrollment process. Obstructive CAD was defined as hemodynamically relevant stenosis, either by visual evaluation (≥80%) or by FFR pressure wire measurement (<0.80) or iwFR (<0.89); TnI, Troponin I; PCI, percutaneous coronary intervention; CABG, coronary artery bypass graft.

In this retrospective observational monocentric study, we analyzed patients with AIS or TIA and elevated serum TnI levels, detected within the first 48 h after hospital admission from a prospectively and consecutively collected database recording patients with AIS and TIA treated at a comprehensive stroke unit (University Hospital Mannheim, <city>Mannheim</city>, Germany); we retrospectively enrolled patients treated between March 2010 and May 2020 (Figure 1). The database records all patients admitted as inpatients due to stroke. All clinical data and technical investigations are recorded and documented according to a standardized acute stroke care protocol. In all AIS patients, the diagnosis was confirmed by either cerebral magnetic resonance imaging (MRI) or computed tomography. TIA was defined as a reversible neurological dysfunction lasting ≤24 h without corresponding acute ischemic lesions in diffusion-weighted MRI (15). Routine assessment on admission to the emergency department included a 12-lead electrocardiogram (ECG) and serum TnI testing.

### Cardiac Workup and Neurological Evaluation

According to the Fourth Universal Definition of MI, MI was suspected in the presence of symptoms of cardiac ischemia, an ECG pattern suggestive of ischemia, such as ST depression or elevation and T wave inversion, or imaging evidence of new regional wall motion abnormalities in a pattern consistent with an ischemic etiology (12). A team of a cardiologist and a neurologist assessed the risk for myocardial ischemia as a consequence of type I MI as well as the potential risk of cerebral bleeding in the case of anticoagulation. This interdisciplinary workup followed a standardized procedure according to an internal algorithm (16). The degree of coronary stenosis was visually evaluated, and if the findings were inconclusive, the stenosis was analyzed for hemodynamic relevance by fractional flow reserve (FFR) or instantaneous wave-free ratio (iwFR). The results were categorized as the absence of CAD, non-obstructive CAD, and obstructive CAD. Non-obstructive CAD was defined as angiographic coronary artery stenosis of ≥50% in any epicardial coronary artery without hemodynamic relevance. Obstructive CAD was defined as hemodynamically relevant stenosis assessed by visual evaluation for severe stenosis (≥80%) or by FFR measurement (<0.80) or iwFR (<0.89). Patients were categorized according to the result of the ICA, and the characteristics of the subgroups were compared. Etiologic classification AIS was performed using the TOAST classification (large-artery atherosclerosis, cardioembolism, small-vessel occlusion) (17). The study protocol was approved by the local medical ethics committee (number 2019-850R).

### Clinical and Laboratory Data

Blood samples were collected on admission to determine TnI and creatine kinase levels, the lipid profile, and renal status. We chose March 2010 as the beginning of the study period because the TnI assay by Siemens Dimension® Vista 1500™ was introduced in our hospital at that time; this assay has a 99th percentile upper reference limit with a limit of detection of 0.015 ng/mL. An elevation of the serum TnI level was defined as a concentration of >0.045 ng/ml. All information regarding clinical data and the results of technical investigations were extracted from the stroke unit database.

### Statistical Analysis

All analyses were performed using SAS (Version 9.4; SAS Institute Inc., Cary, NC, USA). Categorical variables are presented as percentages. Continuous variables, analyzed with an independent sample *t*-test or Mann-Whitney U test, are presented as either the mean ± standard deviation or the median with interquartile range. Multiple logistic regression was used to identify risk factors that predict the absence of CAD, non-obstructive CAD, and obstructive CAD. The following variables were included in a multivariate analysis: sex, age over 65 years, history of CAD, ≥3 cardiovascular risk factors, clinical signs or symptoms of myocardial ischemia, and a left ventricular ejection fraction <40%. The most important variables were selected by stepwise elimination and were assessed for collinearity. The receiver operating characteristic (ROC) curves resulting from these analyses were presented, and the area under the ROC curve (AUC) was calculated. A *p*-value of <0.05 was considered statistically significant.

## Results

In total, 8,322 patients with neuroimaging-confirmed AIS or TIA diagnoses were screened for TnI elevation. A significant elevation of TnI; (>0.045 ng/mL above the 99^th^ percentile) within the first 48 h after admission to the emergency department was found in 2,205 patients (22.5%). The main reasons for exclusion from the final analysis were missing measurements of TnI levels on hospital admission or the absence of an indication for ICA after non-invasive cardiac evaluation. A total of 123 patients (mean age: 71 ± 12 years; 67% male) with AIS and elevated TnI levels who underwent ICA matched the inclusion criteria and were enrolled in the final analysis. A total of 35 (28%) patients were treated with thrombolysis and 8 (7%) underwent mechanical thrombectomy. A comparison of the cardiac status, the cardiac evaluation at admission, the neurological evaluation, risk factors and laboratory values of patients diagnosed with obstructive CAD and those with no or non-obstructive CAD is summarized in Table 1.

**Table 1:** Baseline characteristics.

	**All**	**Non-obstructive coronary artery disease**	**Obstructive coronary artery disease**	***p*-value**
Number of patients	123	72	51	
Age (years)	71 ± 12	70 ± 13	71 ± 11	0.08
Male Sex	82 (67%)	41 (57%)	41 (80%)	<0.01^**^
**Cardiac status**				
History of coronary artery disease	36 (29%)	17 (23%)	19 (37%)	0.14
Atrial fibrillation	34 (28%)	21 (29%)	13 (25%)	0.65
LVEF <40%	35 (29%)	19 (26%)	16 (31%)	0.55
Cardiomyopathy	15 (12%)	11 (15%)	4 (8%)	0.27
Mitral regurgitation	22 (18%)	12 (17%)	10 (20%)	0.74
Aortic stenosis	20 (16%)	10 (14%)	10 (20%)	0.44
Peripheral artery disease	21 (17%)	6 (8%)	15 (29%)	<0.01^**^
**Cardiac evaluation at admission**				
Grace Score >140	61 (50%)	37 (51%)	24 (47%)	0.64
Killip Classification mean ± SD	1.3 ± 2.7	1.4 ± 2.6	1.3 ± 0.7	0.22
Newly diagnosed CAD	62 (50%)	30 (42%)	32 (63%)	<0.05^*^
Newly diagnosed atrial fibrillation	31 (25%)	21 (30%)	10 (20%)	0.23
Signs/symptoms of myocardial ischemia	51 (41%)	18 (35%)	33 (65%)	<0.01^**^
Ischemic ECG pattern	48 (32%)	27 (37%)	21 (41%)	0.85
Angina pectoris	27 (22%)	11 (15%)	16 (31%)	<0.05^*^
Regional wall motion abnormalities	48 (39%)	23 (32%)	25 (49%)	0.07
Hypertensive emergency (syst. ≥180 mmHg)	20 (25%)	17 (24%)	14 (27%)	0.68
**Neurological evaluation**				
TIA	8 (7%)	3 (4%)	5 (10%)	0.25
Stroke insular cortex	19 (15%)	13 (18%)	6 (12%)	0.45
Cardioembolic etiology	78 (63%)	49 (68%)	29 (57%)	0.25
Macroangiopathic etiology	13 (11%)	4 (6%)	9 (18%)	<0.05^*^
Microangiopathic etiology	10 (8%)	5 (7%)	5 (10%)	0.74
NIHSS admission (IQR)	4.0 (1.0–4.0)	4.0 (1.75–8.25)	4.0 (1.0–7.0)	0.5
NIHSS discharge (IQR)	2 (0.0.−4.0)	2 (0.0.−4.0)	2 (0.0–4.25)	0.86
Thrombolysis	35 (28%)	20 (28%)	15 (29%)	0.8
Thrombectomy	8 (7%)	5 (7%)	3 (6%)	0.99
Previous acute ischemic stroke	20 (16%)	11 (15%)	9 (18%)	0.73
**Risk factors**				
Arterial hypertension	86 (70%)	46 (64%)	40 (78%)	0.11
Diabetes mellitus	45 (37%)	23 (32%)	22 (43%)	0.25
Smoking	33 (27%)	16 (22%)	17 (33%)	0.22
Dyslipidemia	58 (47%)	29 (40%)	29 (57%)	0.07
≥3 cardiovascular risk factors	51 (41%)	30 (35%)	21 (55%)	<0.05^*^
CHA_2_DS_2_VASc-Score	4.4 ± 1.8	4.2 ± 1.7	4.9 ± 1.4	<0.05^*^
Pt. <2	18 (14%)	9 (12%)	9 (18%)	0.43
Pt. ≥2	105 (85%)	57 (79%)	48 (94%)	<0.05^*^
Pt. ≥4	90 (73%)	44 (61%)	46 (90%)	<0.001^**^
**Laboratory values**				
Troponin I 1 [ng/ml]	1.3 ± 3.4	0.8 ± 1.6	1.9 ± 4.8	0.98
Creatine kinase [U/l]	304.1 ± 633.0	287.8 ± 459.8	327.1 ± 822.5	0.43
Creatine kinase MB [U/l]	50.2 ± 94.6	38.7 ± 23.6	68.2 ± 148.8	0.07
Creatinine [mg/dl]	1.2 ± 0.7	1.1 ± 0.3	1.3 ± 1.0	0.11
eGFR [ml/min]	60.89 ± 19.2	60.2 ± 18.9	61.9 ± 19.9	0.62
Total - cholesterol [mg/dl]	174.6 ± 63.2	178.0 ± 49.2	169.8 ± 43.8	0.34
LDL - cholesterol [mg/dl]	107.7 ± 37.0	109.4 ± 35.7	105.5 ± 38.9	0.57
HDL - cholesterol [mg/dl]	48.5 ± 17.0	49.9 ± 18.0	46.7 ± 15.6	0.32
Triglycerides [mg/dl]	132.5 ± 63.2	135.4 ± 67.4	128.5 ± 57.1	0.55
HbA1c [%]	6.4 ± 1.4	6.4 ± 1.6	6.2 ± 1.2	0.48

*Variables are presented as mean ± standard deviation median or number (%); SD, standard deviation; IQR, interquartile range; Transient ischemic attack (TIA); National Institutes of Health Stroke Scale (NIHSS) range from 0 to 42 (Data were missing for 4 patients); eGFR, estimated glomerular filtration rate; LDL, low density lipoprotein; HDL, high density lipoprotein; LVEF, left ventricular ejection fraction; Pt., points*.

* *Significant;*

** *Highly significant*.

### Coronary Angiography Findings

CAD was present in 98 (80%) patients; in 62 (51%), CAD was newly diagnosed, while 36 (29%) patients had a history of CAD. Obstructive CAD was found in 51 (41%) patients and was more frequently present in patients with newly diagnosed CAD than in those with a history of CAD (63 vs. 37%; p = 0.03). Thus, ICA finding of obstructive CAD accounts for 2.3% of patients with troponin elevation and 0.6% of all stroke patients. In 4 (3%) patients, ICA revealed the indication for coronary artery bypass graft (CABG) surgery.

### Predictors of Coronary Artery Angiography Findings

The presence of at least one characteristic of acute or previous myocardial ischemia (angina pectoris, ischemic ECG changes, or regional wall motion abnormalities) was more frequent in patients with obstructive CAD compared to those with no or non-obstructive CAD (35 vs. 65%; *p* = 0.006) while only angina pectoris (15 vs. 31%; *p* = 0.03) was significantly more prevalent in patients diagnosed with obstructive CAD. The sensitivity of ischemic ECG pattern (41%; CI 0.29–0.55) or regional wall motion abnormalities (49%; CI 0.36–0.62) for obstructive CAD was low.

Overall, there was a pronounced cardiovascular risk in the study population, with 51 (41%) patients reporting ≥3 cardiovascular risk factors. The frequency of individual risk factors did not significantly differ when comparing the obstructive CAD group to the no or non-obstructive CAD groups; however, the presence of a distinct cardiovascular risk burden (≥3 risk factors) indicated obstructive CAD (35 vs. 55%; *p* < 0.05). High thromboembolic risk according to the CHA_2_DS_2_-VASc score (≥4) was associated with the presence of obstructive CAD (61 vs. 90%; *p* < 0.001). The positive predictive value of obstructive CAD in patients with a high CHA_2_DS_2_-VASc score however was rather weak (0.5; 95% CI 0.4–0.6).

**Figure 2:**
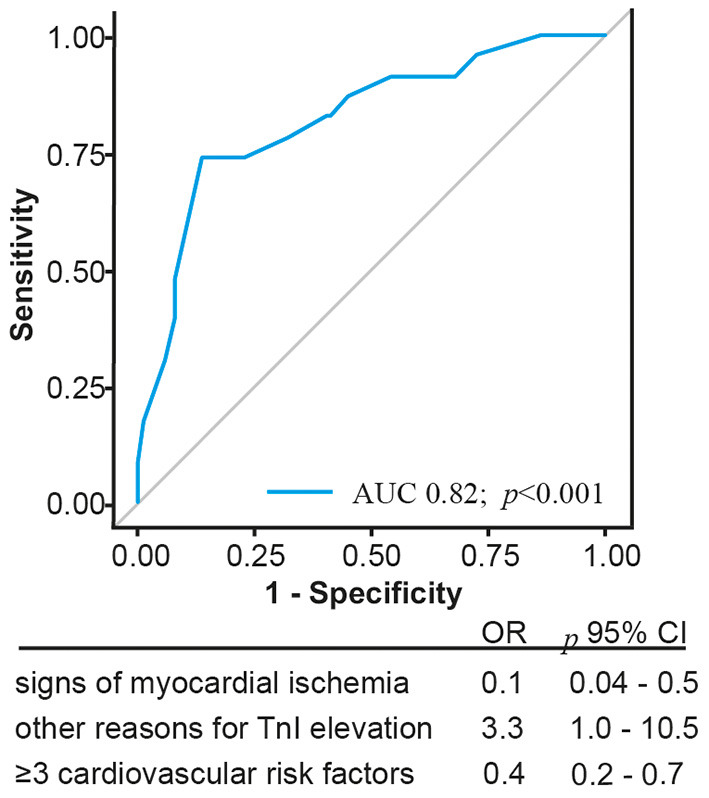
Illustration of the Receiver operating characteristics curve and corresponding Areas of predictors indicating absence of coronary artery disease; CI, confidence interval.

For further evaluation of TnI elevation in patients with no evidence of CAD, the characteristics of this group were compared with those of the CAD group (Table 2). There was a high positive predictive value for exclusion of CAD in imaging evidence of regional wall motion abnormalities (91%; CI 0.80–0.97). A stepwise regression analysis was performed, which defined three factors indicating the absence of obstructive CAD: number of <3 cardiovascular risk factors (≥3 number of cardiovascular risk factors, odds ratio [OR] 0.4; *p* < 0.001), absence of signs or symptoms of MI (signs or symptoms of MI, OR 0.1; *p* < 0.01), and the presence of various causes for TnI elevation (acute severe hypertension, cardiomyopathy, and severe aortic stenosis; OR 3.3; *p* < 0.05). ROC curves of the composite of these predictive associated variables for non-obstructive and excluded CAD are presented in Figure 2. The AUC of this model was 0.82.

**Table 2:** Parameters of patients with no angiographic evidence for CAD.

	**All**	**Coronary artery disease**	**No CAD**	**p-value**
Number of patients	123	98	25	
Signs/symptoms of myocardial ischemia	51 (41%)	38 (39%)	13 (52%)	0.26
Ischemic ECG pattern	48 (32%)	38 (38%)	10 (40%)	0.99
Angina pectoris	27 (22%)	15 (15%)	3 (15%)	0.99
Regional wall motion abnormalities	48 (39%)	44 (45%)	4 (16%)	<0.01^**^
Hypertensive emergency (syst. ≥180 mmHg)	20 (25%)	14 (27%)	17 (24%)	0.68
Severe valve stenosis or insufficiency^†^	37 (30%)	37 (72%)	0 (0%)	
eGFR [ml/min]	60.89 ± 19.2	60.5 ± 18.7	62.5 ± 21.5	0.65
Cardiomyopathy^† †^	15 (12%)	8 (8%)	7 (28%)	<0.05^*^
NIHSS admission	4 (1.0–8.0)	4 (1.5–7.0)	3 (0.25–11.75)	0.94
NIHSS discharge	2 (0.0–4.0)	2 (0.0–6.25)	2 (0.0–4.25)	0.81
Stroke insular cortex	19 (15%)	13 (13%)	6 (24%)	0.22
Thrombolysis	35 (28%)	29 (30%)	6 (24%)	0.8
Thrombectomy	8 (7%)	5 (5%)	3 (12%)	0.2

*SD, standard deviation;*

* *Significant;*

** *Highly significant*,

† *Defined as severe mitral or aortic valve regurgitation and/or stenosis*.

† † *History of ischemic or non-ischemic cardiomyopathy*.

Cardioembolic etiology was identified as the most common causal underlying disease in 63% of all patients. We found a significant association between large-artery atherosclerosis and the presence of obstructive coronary disease (18 vs. 6%, p < 0.05). There was no association between the lesion site or stroke severity and coronary artery findings.

### Alternative Causes for Elevated Troponin I Levels

Non-ischemic, acute, or chronic myocardial injury were considered reasons for elevated TnI levels in the presence of severe valve stenosis or insufficiency in 20 (16%) patients, cardiomyopathy in 15 (12%), impaired renal function (estimated glomerular filtration rate [eGFR] <60 mL/min/1.73 m^2^) in 57 (46%), and hypertensive emergency (systolic blood pressure ≥180 mmHg) in 25 (20%). The presence of alternative causes for elevated TnI levels did not differ in their distribution when comparing the groups without or non-obstructive CAD with the obstructive CAD group. Notably, the presence of previously known cardiomyopathies was associated with no angiographic apparent CAD (12 vs. 28%, *p* < 0.05).

## Discussion

The presented data were collected from a highly selected population in a real-world clinical setting. As these patients were preselected for ICA by means of a high pretest probability for obstructive CAD, a high frequency of obstructive CAD was expected.

Against this expectation, the prevalence of obstructive CAD in our cohort was only 41%. Of these, two-thirds presented with at least one clinical sign of MI (i.e., angina pectoris, ischemic ECG changes, or regional wall motion abnormalities). Of note, ischemic ECG pattern or imaging evidence of regional wall motion abnormalities alone showed a low sensitivity for obstructive CAD. Although the presence of angina pectoris was significantly more common in patients with obstructive CAD (32%), the majority of patients reported no acute cardiac symptoms, which may be attributed to neurological impairment. These results highlight the diagnostic yield of a thorough cardiac examination that includes a detailed anamnesis, 12-lead ECG, and transthoracic echocardiography in all patients presenting with AIS or TIA and elevated TnI levels.

The prospective TRELAS cohort enrolled consecutive AIS patients with troponin elevation. In this unselected cohort, an obstructive CAD was found in 50%, and coronary culprit lesions were present in 24% (11). In our cohort of patients with suspected type I MI, hemodynamically relevant stenosis requiring revascularization was found in only half of patients. This highlights the need for more specific predictors for type I MI in patients with AIS and elevated troponin.

However, the initial diagnostic workup may be compromised by limited diagnostic resources, and diagnostic findings suggesting acute myocardial ischemia may be misleading due to an unknown preexisting myocardial impairment (10). Nevertheless, a known history of CAD was not associated with obstructive CAD. A history of CAD increases the likelihood of type 1 MI, so ICA might have been scheduled more frequently in these patients (18). Due to the necessity to make the indication for ICA in a timely manner, solid predictors for the presence of coronary culprit lesions are urgently needed. If on the other hand, a culprit lesion is considered unlikely, non-invasive imaging techniques should be used for further evaluation of the etiology of the myocardial damage (19).

### Predictors for Obstructive Coronary Artery Disease in Patients With Acute Cerebral Ischemia

Prediction of CAD by the CHA_2_DS_2_-VASc score has been analyzed in the general population and evaluated in patients with AIS, and seems to be associated with asymptomatic CAD (20, 21). In this data set, obstructive CAD was strongly associated with the cumulative number of clinical risk factors (≥3; OR 3.9) and CHA_2_DS_2_-VASc score (≥4 pt.; OR 5.8). Although there is evidence that the CHA2DS2-VASc score is related to severity of CAD, its predictive role for the presence and severity of a CAD beyond the pure summation of cardiovascular risk factors needs further investigation (22).

In subgroups of patients with low cardiovascular risk who have experienced an AIS or TIA but have a corresponding low likelihood of obstructive CAD, an exclusion strategy may be followed based on risk prediction. While unexpectedly, regional wall motion activities failed to significantly indicate obstructive CAD, the specificity of this modality for the exclusion of any CAD was found to be considerable in our cohort. The absence of clinical signs or symptoms of myocardial ischemia and low cardiovascular risk make obstructive CAD unlikely and ICA may be postponed until after neurological recovery. Patients with need for revascularization corresponds to 0.6% of all included patients and (8%) of patients with TnI elevation. However, several comorbidities may cause non-ischemic acute myocardial injury, worsening post-stroke cardiac prognosis (13). The identification of these non-ischemic causes of TnI elevation should complement the diagnostic workup after ruling out type I MI.

We identified a significant association between large-artery atherosclerosis and the presence of obstructive coronary disease. This finding aligns with previous research indicating that the intima and media thickness of the carotid artery is associated with the prevalence of cardiovascular disease and that symptomatic CAD patients have a high prevalence of intra- and extracranial atherosclerotic lesions (23, 24). In nonfatal cerebral infarction, CAD has been shown to be highly predictive of future major vascular events, and this risk especially increased with the extent and severity of coronary and extracranial atherosclerotic disease (25). Vice versa, our data suggest a high prevalence of obstructive CAD in patients whose stroke etiology was classified as atherosclerotic disease of the large vessels. Therefore, AIS patients with symptomatic large-artery atherosclerosis and elevated troponin may represent an especially vulnerable subgroup of stroke patients with risk for obstructive CAD.

## Limitations

First, our study is based on retrospective data analysis. In clinical practice, a decision to perform ICA is determined not only by cardiac and neurological findings but also by the patient's preference or other comorbidities, resulting in a selection bias. Second, due to the study design resulting in a highly selected cohort, patients with a high pretest probability of CAD were included in the final analysis. Due to severe neurological disability in some patients, ICA could not be performed. Third, some degree of overlap in the etiology of TnI elevation, such as the combination of non-ischemic myocardial injury and type II MI, is probable. Not all included patients underwent extended diagnostics, such as cardiac MRI or myocardial scintigraphy, to detect myocardial ischemia. Therefore, no conclusions can be drawn as to the cause of non-coronary myocardial damage in these patients. Fourth, the complex treatment of patients with AIS may require thrombectomy, thrombolysis, or intensive care, leading to varying sampling intervals of TnI levels. Thus, TnI kinetics and the quantity of delta TnI for these patients were not reflected in our data.

Owing to the high pretest probability of obstructive CAD in our study design, our models did not achieve sufficient measures of separability. The validation of risk factors and establishment of a risk prediction instrument should be based on prospective trials with uniform study protocols, such as the PRAISE study (NCT: 03609385) (26).

## Conclusion

Only 40 % of AIS and TIA patients with clinically suspected type I MI presented obstructive CAD in our real-world cohort. The cumulation of several cardiovascular risk factors and clinical signs of MI were predictive. Therefore, the extent of cardiovascular risk should be weighed against alternative causes for TnI elevation and more specific predictors for an obstructive CAD are urgently needed. AIS patients with large-artery atherosclerosis and elevated troponin may represent an especially vulnerable subgroup of stroke patients with risk for obstructive CAD.

## Data Availability Statement

The original contributions presented in the study are included in the article/supplementary material, further inquiries can be directed to the corresponding author.

## Ethics Statement

The studies involving human participants were reviewed and approved by Ethics Committee II of the University Heidelberg, Medical Faculty Mannheim. The patients/participants provided their written informed consent to participate in this study.

## Author Contributions

MK designed and conceptualized the study, analyzed and interpreted the data, and drafted the manuscript. AK had a major role in the acquisition of data, interpreted the data, and revised the manuscript to intellectual content. CF, IM, MK, MiB, IA, MaB, and KS interpreted the data and revised the manuscript to intellectual content. SH did the statistical analysis and revised the manuscript to intellectual content. SB designed and conceptualized the study, major role in the acquisition of data, and revised the manuscript for intellectual content. All authors contributed to the article and approved the submitted version.

## Conflict of Interest

The authors declare that the research was conducted in the absence of any commercial or financial relationships that could be construed as a potential conflict of interest.

## Publisher's Note

All claims expressed in this article are solely those of the authors and do not necessarily represent those of their affiliated organizations, or those of the publisher, the editors and the reviewers. Any product that may be evaluated in this article, or claim that may be made by its manufacturer, is not guaranteed or endorsed by the publisher.

## Abbreviations

AIS: Acute ischemic stroke
CABG: Coronary artery bypass graft
CAD: Coronary artery disease
CI: Confidence interval
ECG: Electrocardiogram
eGFR: Estimated glomerular filtration rate
FFR: Fractional flow reserve
iwFR: Instantaneous wave-free ratio
ICA: Invasive coronary angiography
MI: Myocardial infarction
MRI: Magnet resonance imaging
OR: Odds ratio
TIA: Transient ischemic attack
TnI: Troponin I.

## Appendix

1) **Hypertension** is defined as systolic blood pressure >140 mmHg, diastolic blood pressure >90 mmHg, or the use of antihypertensive medication.
2) **Dyslipidemia** is defined as a total cholesterol level >240 mg/dL (>6.2 mmol/L) or the use of cholesterol-lowering drugs to reduce cardiovascular risk, according to ESC guidelines.
3) **Diabetes mellitus** is defined as fasting plasma glucose ≥7.0mmol/L (126 mg/dL) or 2-h plasma glucose ≥11.1 mmol/L (200 mg/dL).
4) **Aortic valve stenosis:** patients were considered to have aortic valve stenosis if severity of the stenosis was graded by echocardiography to be not less than moderate.
5) **Mitral regurgitation** patients were considered to have mitral regurgitation if echocardiographic confirmed diagnosis of not less than moderate graded mitral regurgitation was present.
6) The **CHA2DS2-VASc score** was calculated based on the 2014 AHA/ACC/HRS guidelines, as a tool of choice in stroke risk assessment.
7) **Grace-Score:** The Global Registry of Acute Coronary Events score (GRACE), developed by a multinational registry, was used according to the ESC guidelines with a cut-off level of >140 points.
8) The **Killip classification** was assessed based on the Killip and Kimball criteria.
9) An **ischemic ECG pattern** was classified as ST segment depression or elevation >1 mV in the limb leads or T wave inversion in any lead.
10) **Regional myocardial wall motion abnormalities**, detected by echocardiography, are based on the visually observed wall thickening and endocardial motion of the myocardial segments during the myocardial contraction. In stress echocardiography, regional wall motion abnormality may reveal hemodynamical relevant coronary artery stenosis.
